# *In Vitro* and *in Vivo* Neuroprotective Effects of Walnut (Juglandis Semen) in Models of Parkinson’s Disease

**DOI:** 10.3390/ijms17010108

**Published:** 2016-01-15

**Authors:** Jin Gyu Choi, Gunhyuk Park, Hyo Geun Kim, Dal-Seok Oh, Hocheol Kim, Myung Sook Oh

**Affiliations:** 1 Department of Life and Nanopharmaceutical Sciences, Graduate School, Kyung Hee University, 26 Kyungheedae-ro, Dongdaemun-gu, Seoul 130-701, Korea; choijg2002@khu.ac.kr (J.G.C.); uranos5@kiom.re.kr (G.P.); 2 Department of Oriental Pharmaceutical Science, College of Pharmacy and Kyung Hee East-West Pharmaceutical Research Institute, Kyung Hee University, 26 Kyungheedae-ro, Dongdaemun-gu, Seoul 130-701, Korea; kimhyogeun@khu.ac.kr; 3 Division for Medical Research, Korea Institute of Oriental Medicine, 1672 Yuseong-daero, Yuseong-gu, Daejeon 34054, Korea; dsoh@kiom.re.kr; 4 Department of Herbal Pharmacology, College of Korean Medicine, Kyung Hee University, 26 Kyungheedae-ro, Dongdaemun-gu, Seoul 130-701, Korea; hckim@khu.ac.kr

**Keywords:** Juglandis Semen, walnut, neuroprotection, monoamine oxidase, Parkinson’s disease, dopaminergic neuron

## Abstract

Monoamine oxidase (MAO) catalyzes the oxidative deamination of monoamines including dopamine (DA). MAO expression is elevated in Parkinson’s disease (PD). An increase in MAO activity is closely related to age, and this may induce neuronal degeneration in the brain due to oxidative stress. MAO (and particularly monoamine oxidase B (MAO-B)) participates in the generation of reactive oxygen species (ROS), such as hydrogen peroxide that are toxic to dopaminergic cells and their surroundings. Although the polyphenol-rich aqueous walnut extract (JSE; an extract of Juglandis Semen) has been shown to have various beneficial bioactivities, no study has been dedicated to see if JSE is capable to protect dopaminergic neurons against neurotoxic insults in models of PD. In the present study we investigated the neuroprotective potential of JSE against 1-methyl-4-phenylpyridinium (MPP^+^)- or 1-methyl-4-phenyl-1,2,3,6-tetrahydropyridine (MPTP)-induced neurotoxicities in primary mesencephalic cells and in a mouse model of PD. Here we show that JSE treatment suppressed ROS and nitric oxide productions triggered by MPP^+^ in primary mesencephalic cells. JSE also inhibited depletion of striatal DA and its metabolites *in vivo* that resulted in significant improvement in PD-like movement impairment. Altogether our results indicate that JSE has neuroprotective effects in PD models and may have potential for the prevention or treatment of PD.

## 1. Introduction

Parkinson’s disease (PD) is a common neurodegenerative disease whose main symptom is movement impairment (e.g., rigidity, resting tremor, hypokinesia, bradykinesia, and postural instability) due to degeneration of the brain nigrostriatal system. This process is characterized by the progressive loss of dopaminergic neurons in the substantia nigra pars compacta (SNpc) and is accompanied by depletion of striatal dopamine (DA) and its metabolites, including 3,4-dihydroxyphenylacetic acid (DOPAC) and homovanillic acid (HVA) [1,2]. Current PD treatment is symptomatic and primarily involves pharmacological therapies including DA replacement with synthetic DA agonists such as levodopa and with drugs that increase the DA supply by inhibiting catechol-*O*-methyl transferase and monoamine oxidase B (MAO-B) [3].

MAO-B, found primarily in non-neuronal brain cells, such as astrocytes and radial glia, catalyzes the oxidative deamination of biogenic and xenobiotic amines and plays an important role in the metabolism of DA in the central nervous system [4,5]. Age-related elevation of MAO-B expression may trigger neuronal damage in the brain, due to generating oxidative stress, and thus, producing reactive oxygen species (ROS) such as hydrogen peroxide (H_2_O_2_). High levels of MAO-B positive astrocytes are concentrated in the SNpc, a region of the brain specifically impacted in PD. MAO-B activated ROS can cause cell damage in surroundings as well as in neurons [6], and neurons are particularly vulnerable to the oxidative stress [7]. MAO-B levels increased twofold in the SNpc in PD models and this correlates well with the percentage of selective dopaminergic cell loss in the SNpc [7,8]. In fact, it is known that MAO-B inhibitors diminish DA degradation and prevent the formation of neurotoxic DA metabolites and ROS. As a result, this type of agent is currently used in the symptomatic treatment of PD due to their neuroprotective effects [9,10,11,12].

The neurotoxin 1-methyl-4-phenyl-1,2,3,6-tetrahydropyridine (MPTP), is routinely used to induce PD-like neurodegeneration in experimental animal models [13]. The toxic effects of MPTP actually result from 1-methyl-4-phenylpyridinium (MPP^+^) which is produced by MAO-B when the enzyme oxidizes MPTP into MPP^+^ [13,14]. MPP^+^ is produced in the astrocytes of the brain and is taken into dopaminergic neurons by dopamine transporters [15,16]. It accumulates in the mitochondria and inhibits Complex I of the cell process of respiration [17,18]. With this energy deficiency, it triggers serially dopaminergic neuronal cell death [19]. Moreover, the occurrence of oxidative stress is directly caused by MPP^+^ toxicity [20]. It is reported that MPP^+^ and nicotinamide adenine dinucleotide (NADH) dehydrogenase in the mitochondria are closely associated with H_2_O_2_-mediated hydroxyl radical (·OH) generation when MPP^+^ may contribute via the Fenton reaction when MPP^+^ contributes to the release of Fe^2+^ [20,21]. These toxic substances have been viewed as primary pathological factors of PD [20,22,23].

Juglandis Semen (JS; walnut) is a seed of *Juglans regia* L. of the Juglandaceae family and it is commonly consumed and also used as a medicinal herb [24,25]. Previous studies reported that polyphenol-rich extract of JS inhibited low-density lipoprotein oxidation [26] and promoted osteoblastic activity in human aorta endothelial cells [24]. JS also protected against cyclophosphamide-induced biochemical toxicity in mouse liver and kidney [27]. Caffeic acid which is rich in JS significantly inhibited the MAO-B activity in rat C6 astrocyte cells, and its phenethyl ester derivative protected against 6-hydroxydopamine-induced neuronal degeneration [28,29]. In previous studies on the neuropharmacological effects of JS, reduction of neuroinflammatory factors such as nitric oxide (NO), tumor necrosis factor-α and inducible nitric oxide synthase in lipopolysaccharide (LPS)-stimulated mouse microglial cell have been shown [30]. JS also exhibited anticonvulsant and neuroprotective activities in pentylenetetrazol-induced seizures [31]. Also, JS and ω-3 fatty acids inhibited hippocampal cell death by LPS-mediated calcium dysregulation [32] and improved amyloid-beta fibril-induced memory deficits in a transgenic mouse model of Alzheimer’s disease (AD) [33,34]. However, there has been no report on the protective effects of JS on dopaminergic neurons exposed to neurotoxic stimuli.

In the present study, we evaluated the effects of JS on MAO-B activity and its protective effect against MPP^+^ or MPTP-induced toxicity *in vitro* and *in vivo*, respectively. We have also investigated the effects of JS against MPP^+^-induced ROS and NO generation due to elevation of MAO-B.

## 2. Results

### 2.1. Determination of Caffeic Acid in Extract of Juglandis Semen (JSE) Using High Performance Liquid Chromatography (HPLC)

**Figure 1:**
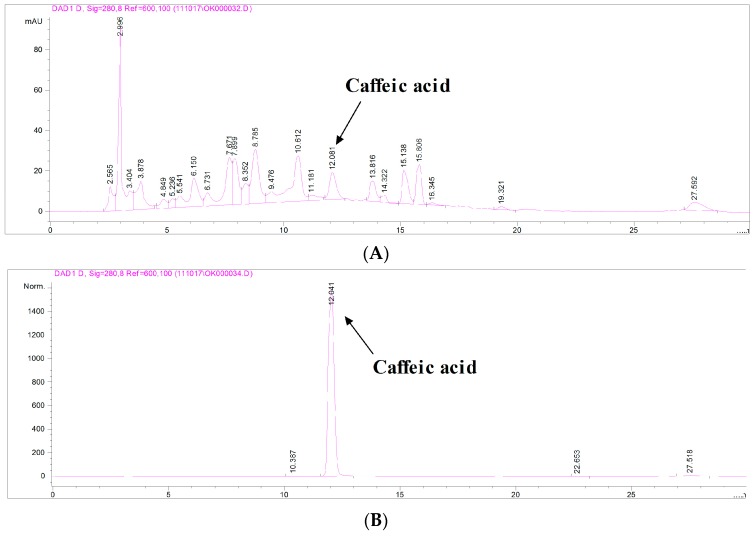
High performance liquid chromatography (HPLC) analysis of caffeic acid in JSE (Extract of Juglandis Semen). HPLC chromatogram of JSE (**A**) and external standard caffeic acid (**B**).

The retention times of reference standard caffeic acid and caffeic acid in JSE were found to be 12.04 and 12.08 min, respectively (Figure 1). The content of caffeic acid in JSE was 1.00% *w*/*w* as determined from the linear regression equation of the calibration graph for this compound.

### 2.2. Effects of JSE on Monoamine Oxidase B (MAO-B) Activity in Vitro

**Figure 2:**
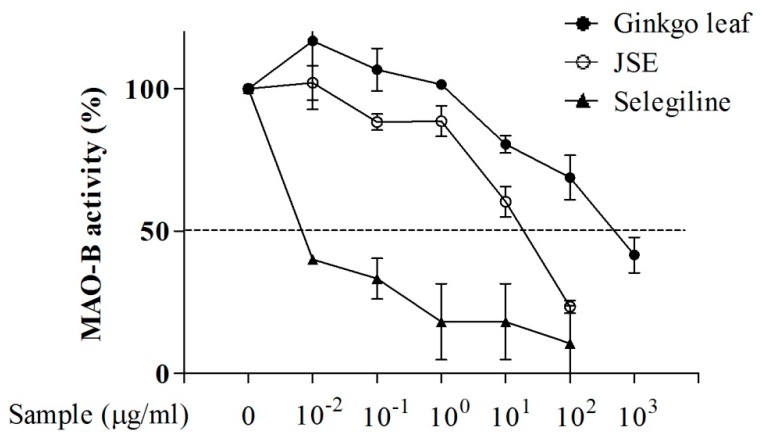
Inhibitory effect of JSE on MAO-B activity *in vitro*. JSE and positive controls (Ginkgo leaf extract and selegiline) inhibited Monoamine Oxidase B (MAO-B) activity in a concentration-dependent manner from 0, 0.01, 0.1, 1, 10, 100 μg/mL.

To investigate the inhibitory effect of JSE on MAO-B activity, rat liver homogenate MAO was used after purification and isolation from mitochondria. Selegiline, a selective irreversible inhibitor of MAO-B, is widely used in the treatment of PD, and Ginkgo leaf extract produces reversible inhibition of rat brain MAO [35]. As shown in Figure 2, similarly to the positive controls (ginkgo leaf extract and selegiline) JSE inhibited MAO-B activity with an IC_50_ of 42.59 ± 4.25 μg/mL. For ginkgo extract the IC_50_ was 710.32 ± 2.36 μg/mL and for selegiline this value was found to be 0.016 ± 1.06 μg/mL) (Figure 2).

### 2.3. Effects of JSE on 1-Methyl-4-phenylpyridinium (MPP^+^)-Induced Reactive Oxygen Species (ROS) and NO Generation in Vitro

**Figure 3:**
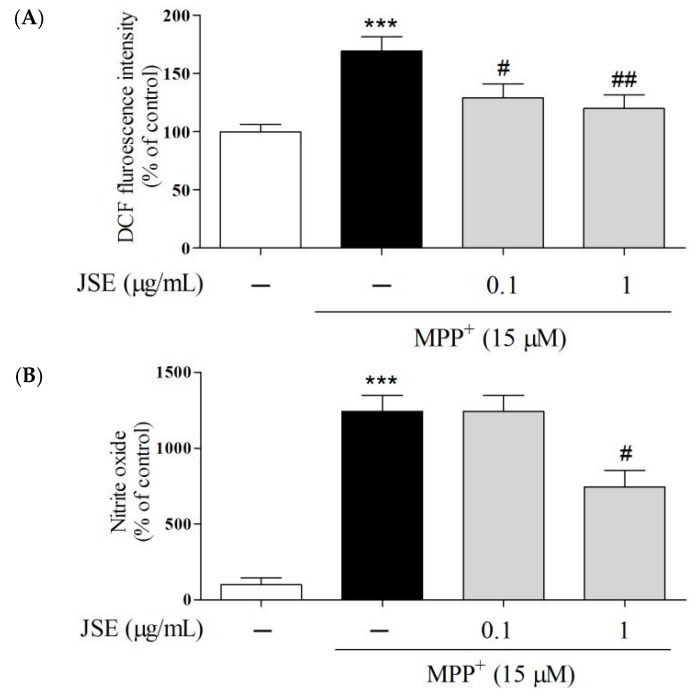
Effect of JSE on MPP^+^-induced ROS and NO generations *in vitro*. 2,7-dichlorofluorescein (DCF) fluorescence intensity was measured after exposure of primary mesencephalic cells to 15 µM 1-methyl-4-phenylpyridinium (MPP^+^) for 1 h, followed by 25 µM 2,7-dichlorodihydrofluorescein diacetate (H_2_DCF-DA) for 30 min (**A**); Extracellular NO production in JSE-pretreated (23 h) primary mesencephalic cells before 15 µM MPP^+^ treatment was assayed by measuring NO in the supernatant using the Griess reagent (**B**). Values were expressed as means ± SEM. *** *p* < 0.001 *vs.* the control group; ^#^ *p* < 0.05 and ^##^ *p* < 0.01 *vs.* the MPP^+^-only treated group.

To investigate whether JSE protected against MPP^+^-induced intracellular ROS and extracellular NO generation, 2,7-dichloro-dihydro-fluorescein diacetate (DCFH-DA) fluorescent dye and Griess reagent were used, respectively. MPP^+^ exposure (15 μM) led to significant intracellular ROS elevation in primary rat mesencephalic neuron/glia mixed cells (169% ± 10%, *p* < 0.01) as compared to the control group. Pre-treatment with JSE at 0.1 and 1 µg/mL inhibited intracellular ROS generation (129% ± 10% and 120% ± 12%; Figure 3A) in the MPP^+^ only group. Also, in the extracellular NO study, MPP^+^ exposure (15 μM) led to significant extracellular NO elevation in primary rat mesencephalic neuron/glia mixed cells (1244% ± 104%, *p* < 0.001) as compared to the control group. Pre-treatment with JSE at 0.1 μg/mL (1241% ± 107%, no significant) and 1 µg/mL (746% ± 108%, *p* < 0.05) inhibited extracellular NO generation compared to the MPTP only group (Figure 3B).

### 2.4. Effects of JSE against MPP^+^ or 1-Methyl-4-phenyl-1,2,3,6-tetrahydropyridine (MPTP)-Induced Dopaminergic Cell Death in Vitro and in Vivo

**Figure 4:**
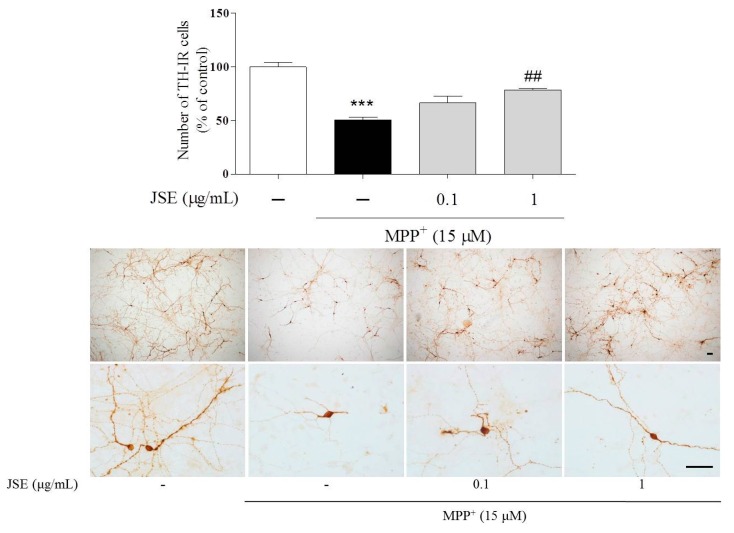
Effects of JSE against MPP^+^-induced neurotoxicity *in vitro*. Rat primary mesencephalic cells were treated with JSE for 1 h, followed by MPP^+^ exposure for 23 h. The number of anti-tyrosine hydroxylase -immunoreactive (TH-IR) neurons was measured after cells were fixed and stained. Representative images are shown. Scale bar = 100 µm. Values were expressed as means ± SEM. *** *p* < 0.001 *vs.* the control group; ^##^ *p* < 0.01 *vs.* the MPP^+^-only treated group.

In our *in vitro* study to examine the protective effects of JSE against MPP^+^-induced toxicity in primary rat mesencephalic neuron/glia mixed cells we measured anti-tyrosine hydroxylase-immunoreactive (TH-IR) cell bodies after JSE treatment with or without MPP^+^ toxicity. In control cultures, we observed 400 to 600 TH-positive cells per coverslip. MPP^+^ treatment resulted in a reduced number of TH-IR neurons (51% ± 2%, *p* < 0.001) as compared to the control group. However, JSE treatment at 0.1 μg/mL (67% ± 5%, no significant) and 1 μg/mL (78% ± 1%, *p* < 0.01) reduced dopaminergic cell loss induced by MPP^+^ toxicity as compared to the MPP^+^ only group. Representative photomicrographs are shown (Figure 4).

**Figure 5:**
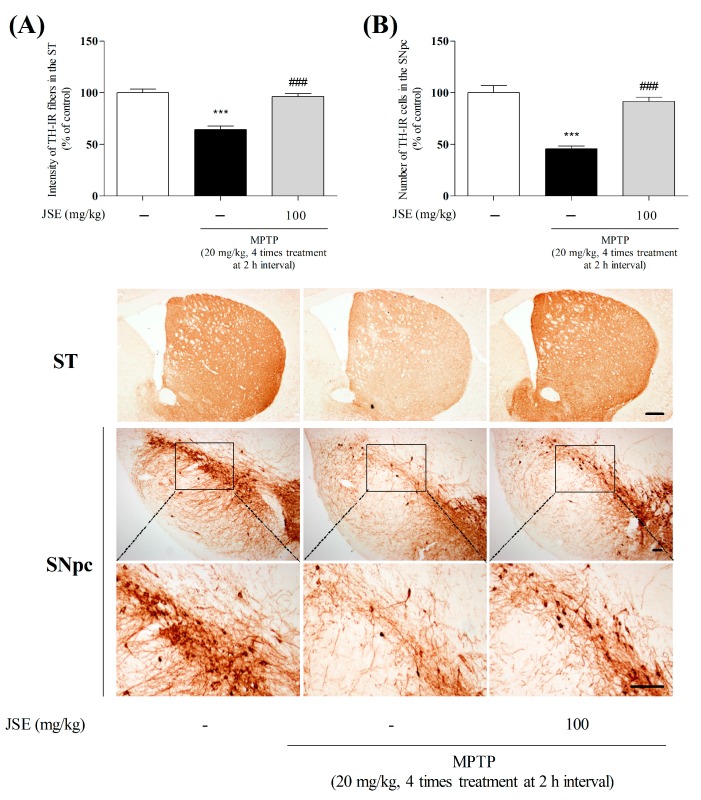
Effects of JSE against 1-methyl-4-phenyl-1,2,3,6-tetrahydropyridine (MPTP)-induced neurotoxicity *in vivo*. Dopaminergic fibers and neurons were visualized by anti-tyrosine hydroxylase (TH) immunostaining. The optical density in the striatum (ST) (**A**) was measured and the number of TH-positive neurons in the substantia nigra pars compacta (SNpc) (**B**) was counted. Representative photomicrographs are shown. The squares are concentration areas of TH-positive neurons in the SNpc. Scale bar = 100 µm. Values were expressed as means ± SEM. *** *p* < 0.001 *vs.* the control group; ^###^ *p* < 0.001 *vs.* the MPTP-only treated group.

In *in vivo* study, to confirm the protective effects of JSE on dopaminergic damage induced by MPTP, we performed TH-IHC (immunohistochemistry) in the striatum (ST) and SNpc. TH-IR fibers and cells were reduced in the MPTP group as compared to the control group (64% ± 5%, *p* < 0.001 and 46% ± 5%, *p* < 0.001). However, JSE + MPTP treatment significantly reduced the loss of fibers and dopaminergic neurons (96% ± 1%, *p* < 0.001 and 91% ± 5%, *p* < 0.001) as compared to the MPTP only group. Representative photomicrographs are shown in Figure 5.

### 2.5. Effects of JSE on MAO Activity in Vivo

**Figure 6:**
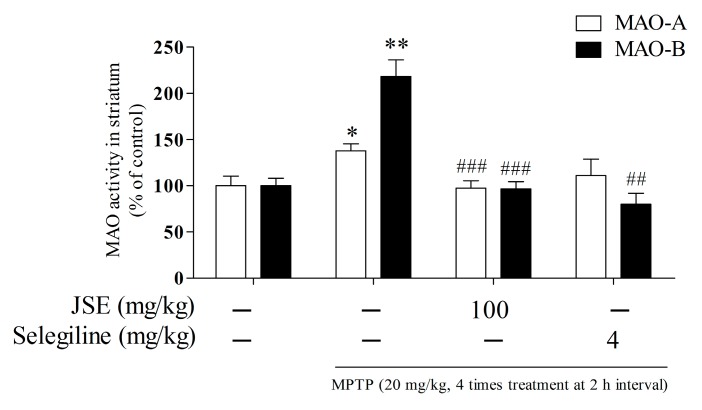
Effect of JSE on MAO activity in *in vivo*. Monoamine oxidase A and B (MAO-A and B) activities in ST of mouse brain were measured by an MAO kit. Values were expressed as means ± SEM. * *p* < 0.05 and ** *p* < 0.01 *vs.* the control group; ^##^ *p* < 0.01 and ^###^ *p* < 0.001 *vs.* the MPTP-only treated group.

To investigate the *in vivo* inhibitory effect of JSE on MPTP-induced monoamine oxidase A and B (MAO-A and B) activities, we measured MAO-A and B activities using an assay kit in the ST of mouse brain. MPTP treatment (20 mg/kg, *i.p.* 4 times treatment at 2 h interval) resulted in increased MAO-A and B activities (138% ± 8%, *p* < 0.05 and 218% ± 18%, *p* < 0.01) as compared to the control group. However, JSE treatment at 100 mg/kg (97% ± 8%, *p* < 0.001 and 97% ± 8%, *p* < 0.001) or selegiline treatment at 4 mg/kg (111% ± 18% and 80% ± 12%, *p* < 0.001) inhibited MAO-A and B activities induced by MPTP toxicity as compared to the MPTP only group (Figure 6).

### 2.6. Effects of JSE against 1-Methyl-4-phenyl-1,2,3,6-tetrahydropyridine (MPTP)-Induced DA Availability in the ST of Mouse Brain

**Figure 7:**
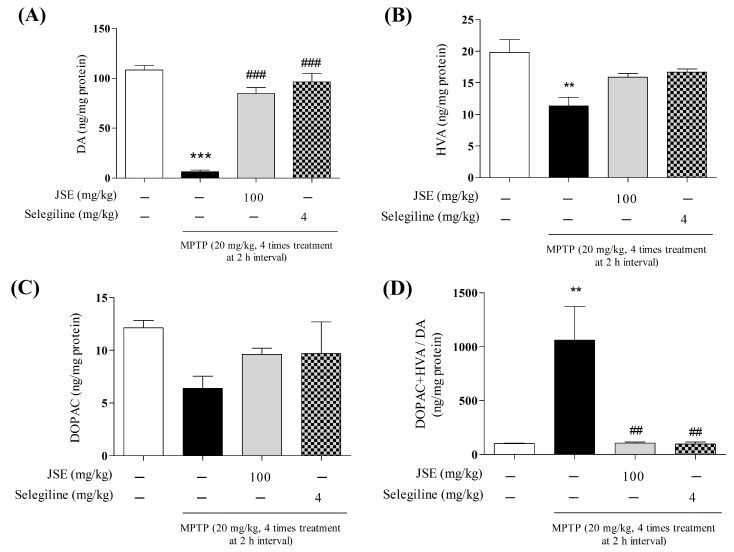
Effect of JSE on MPTP-induced changes neurotransmitter levels in the striatum (ST). JSE (100 mg/kg/day, dissolved in normal saline) was orally administered for six days. After 2 h for third day administration, MPTP (20 mg/kg) dissolved in saline was injected four times. Striatal levels of dopamine (DA) (**A**); homovanillic acid (HVA) (**B**); 3,4-dihydroxyphenylacetic acid (DOPAC) (**C**); and (DOPAC + HVA)/DA (**D**) were determined via HPLC analysis. Values were expressed as means ± SEM. ** *p* < 0.01 and *** *p* < 0.001 *vs.* the control group; ^##^ *p* < 0.01 and ^###^ *p* < 0.001 *vs.* the MPTP-only treated group.

To examine the protective effects of JSE against MPTP on the dopaminergic system, we measured striatal DA and its metabolites using an HPLC-based assay with electrochemical detection. As shown in Figure 7, MPTP administration produced significant depletion of DA (6.46 ± 1.57 ng/mg protein, *p* < 0.001 relative to the control group) and of its metabolites DOPAC (6.40 ± 1.16 ng/mg protein, *p* < 0.001 relative to the control group), HVA (11.32 ± 1.38 ng/mg protein, *p* < 0.001). On the other hand, selegiline or JSE in the MPTP-treated groups prevented the changes in DA (84.92 ± 6.06 and 96.47 ± 8.35 ng/mg protein), DOPAC (9.60 ± 0.59 and 9.69 ± 3.01 ng/mg protein), and HVA (15.89 ± 0.60 and 16.68 ± 0.51 ng/mg protein). Moreover, MPTP toxicity resulted in increased (DOPAC + HVA)/DA ratio was 1061.03 ± 312.92 ng/mg protein due to MPTP toxicity. Selegiline or JSE treatment, however, counteracted the harmful effect of MPTP, as (DOPAC + HVA)/DA was merely 104.12 ± 10.91 and 97.25 ± 18.84 ng/mg protein, respectively.

### 2.7. Effects of JSE against MPTP-Induced Locomotor Ataxia

**Figure 8:**
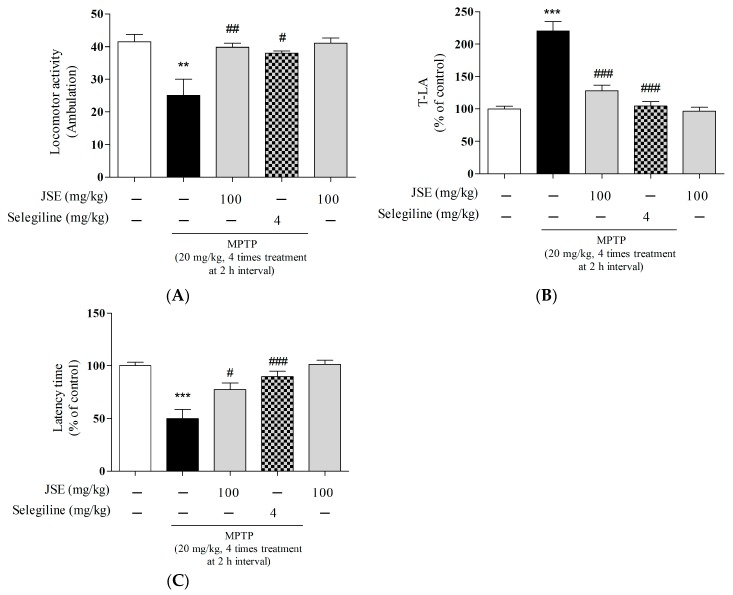
Effect of JSE on MPTP-induced movement impairment in mice. JSE (100 mg/kg/day, dissolved in normal saline) was orally administered for six days. After 2 h for third day administration, MPTP (20 mg/kg) dissolved in saline was intraperitoneally injected four times. Three days after the last injection, ambulation on the locomotor activity was recorded (**A**); The time needed for the mice to climb down and place all four feet on the floor was recorded with a 30 s cut-off limit (**B**); Seven days after MPTP injection, latency time on the rotating rod was recorded with a 300 s cut-off limit (**C**). Values were expressed as means ± SEM. ** *p* < 0.01 and *** *p* < 0.001 *vs.* the control group; ^#^ *p* < 0.05, ^##^ *p* < 0.01, and ^###^ *p* < 0.001 *vs.* the MPTP-only treated group.

To examine the effect of JSE on MPTP-induced poor motor coordination, a locomotor activity test was performed [36]. We found that MPTP administration significantly decreased the ambulation to 25.10 ± 4.98, *p* < 0.001 on day seven, as compared with the control. However, ambulation was significantly increased in the MPTP + selegiline and MPTP + JSE group to 39.80 ± 1.25, *p* < 0.05 and 38.01 ± 0.70, *p* < 0.001, respectively, on day seven, as compared to the MPTP only group (Figure 8A).

### 2.8. Effects of JSE against MPTP-Induced Movement Impairment in the Pole Test

To determine the effect of JSE on MPTP-induced bradykinesia, a pole test was performed on day seven after MPTP injection [37]. T-LA was significantly prolonged to 220% ± 12%, *p* < 0.001 on day seven as compared to the control group. However, T-LA was significantly shortened in the MPTP + selegiline and MPTP + JSE groups to 139% ± 10%, *p* < 0.001 and 104% ± 6%, *p* < 0.001 on day seven, as compared to MPTP alone (Figure 8B).

### 2.9. Effects of JSE against MPTP-Induced Movement Impairment in the Rotarod Test

To examine the effect of JSE on MPTP-induced poor motor coordination and postural balance, a rotarod test was performed [38]. We found that MPTP administration significantly decreased the retention time to 50% ± 8%, *p* < 0.001 on day seven, as compared to the control group. However, retention times were significantly increased in the MPTP + selegiline and MPTP + JSE groups to 8% ± 5%, *p* < 0.001 and 90% ± 4%, *p* < 0.001, respectively, on the seventh day, as compared to the MPTP only group (Figure 8C).

## 3. Discussion

In the present study, we evaluated the *in vivo* potential of JSE to prevent parkinsonian neurodegeneration. We showed that JSE treatment inhibited MPP^+^-induced oxidative and nitrosative stress, thus, prevented mesencephalic neuronal cell death *in vitro*. JSE also protected dopaminergic neurons and prevented the reduction of striatal DA and its metabolites in mice exposed to the neurotoxic effect of MPTP. Moreover, JSE treatment inhibited MPTP-induced PD-like symptoms, including poor ambulation, bradykinesia, motor coordination, and postural balance.

Clinically, the commonly used medications to treat motor symptoms in PD patients are levodopa, dopamine agonists and MAO-B inhibitors depending on the disease stage [39,40]. Selegiline, which is one of MAO-B inhibitors, has especially been used for the treatment of early-stage PD [41,42,43]. MAO-B inhibitors help to block the breakdown of DA in the brain, make more DA available and, thus, reduce some of the motor symptoms [44,45]. Recently, Zhao *et al.* found that selegiline directly inhibited MPTP-induced motor dysfunction through inhibiting the glial cell-derived neurotrophic factor. They have also shown that dopaminergic cell death was also inhibited via downregulating of the Bax/Bcl2 pathway related to neurodegeneration [44]. In addition, MAO inhibitors including selegiline upregulated the activities of antioxidant enzymes and glutathione content in the nigrostriatum [46].

To explore the effects of JSE on MAO-B inhibition via antioxidant mechanism, we measured MAO-B activity and ROS/NO levels. MAO-B naturally increases with age, especially after 50 years of age, and is closely related to the initiation or progression of PD [7,47,48]. DA is oxidized by MAO-B-generated ROS through DA quinone formation to stable dopaminochrome (DACHR), ultimately leading to mitochondrial Complex I dysfunction [5,49,50]. The oxidative radical in mitochondria is increased since DACHR shows the high affinity for molecular oxygen [51]. ROS-mediated DA oxidation is also involved in microglial activation and dopaminergic cell death in the substantia nigra [51]. Besides, oxidative stress-mediated dopaminergic cell death is a major characteristic in toxin-induced PD models [52,53]. In this study, JSE, the water-soluble extract of JS, showed the excellent antioxidant activity of ROS/NO generation and MAO-B inhibitory activity *in vitro* compared to ginkgo leaf extract that is a known natural MAO-B inhibitor [54]. Then, we evaluated whether JSE inhibited the activity of MAO-A as well as MAO-B in the ST of mouse brain. It has been reported that MAO-A regulated cellular signaling systems, especially extracellular signal-regulated kinases/nuclear factor-kappaB pathway and induced cell apoptosis in neurodegenerative disorders, including PD and AD [55,56,57]. Our results showed that JSE significantly inhibited MPTP-induced both striatal MAO-A and B activation *in vivo*. We also investigated whether MAO-B and anti-glial fibrillary acid protein (GFAP) were co-localized in the SNpc of mouse brain in order to confirm the inhibitory effect of JSE on MPTP-induced MAO-B elevation in astrocytes. Our data showed that JSE treatment inhibited MAO-B fluorescence intensity induced by MPTP toxicity, as compared to the control group (Figure S1). JSE also protected against the MPTP-induced loss of TH-positive dopaminergic neurons striatal MAO-A and MAO-B activations *in vivo*. Taken together, JSE showed protective effects against MPTP-induced neurotoxicity by involving MAO inhibition.

To investigate the protective effects of JSE against MPTP-induced neuronal toxicity in the dopaminergic system, we measured striatal DA and its metabolites DOPAC and HVA. DA is a catecholamine synthesized in the brain from the amino acid tyrosine that is metabolized to dihydroxyphenylalanine (DOPA) by the TH [58]. DOPA is then metabolized to DA via aromatic amino acid decarboxylase [50,58]. DA is mainly metabolized by MAO-B and catechol-*O*-methyltransferase [50,58]. Therefore, inhibition of these enzymes would decrease DA metabolism and result in increased levels of DA in the brain [59,60]. Similarly to DA, the level of DA metabolites has also been reported to decrease in PD patients [61]. This study demonstrated that JSE treatment raised the level of not only DA but its metabolites as opposed to MPTP alone. These findings suggest that the MAO inhibitory activity by anti-oxidative effects of JSE may partly prevent the DA loss.

Finally, to evaluate the neuroprotective effect of JSE in ethology, we investigated whether JSE improved movement impairment in a MPTP-induced PD mouse model using locomotor activity [59], pole test [62,63] and rotarod test [64]. The loss of dopaminergic neurons is associated with an onset of motor symptoms and there is a direct relationship between the extent of DA loss and motor dysfunction [63,65]. Our study showed that JSE treatment significantly improved MPTP-induced PD-like movement problems.

Recent studies on neuroprotective effects of walnut showed that a diet supplement of walnut has neuroprotective effects in MPTP mice; the polyphenol-rich walnut ameliorated memory impairment in transgenic mice of AD, and walnut extract protected against amyloid-beta toxicity *in vitro* [66,67,68]. These previous studies together with our results presented here suggest that JSE may be a viable treatment option for neurodegenerative conditions, including PD, although further studies are needed to elucidate the full. Further studies of the active compounds of JSE will be needed for the mode of action, human basal metabolic rate, blood–brain barrier permeable and individual side effects in various experimental models.

## 4. Experimental Section

### 4.1. Chemicals

Minimal essential medium (MEM), and fetal bovine serum (FBS) were purchased from Hyclone Laboratories, Inc. (Logan, UT, USA). Rabbit polyclonal anti-tyrosine hydroxylase (TH) and rabbit monoclonal anti-glial fibrillary acid protein (GFAP) were purchased from Millipore Bioscience Research (Bedford, MA, USA). Goat polyclonal anti-MAO-B was purchased from Santa Cruz Biotechnology (Santa Cruz, CA, USA). Biotinylated anti-rabbit antibody, streptavidin-Alexa 488, goat anti-rabbit IgG Cy3 conjugate Alexa 594, and avidin-biotin complex (ABC) were purchased from Vector Laboratories, Inc. (Burlingame, CA, USA). MPTP, glucose, glutamine, poly-l-lysine (PLL), and 2,7-dichloro-dihydro-fluorescein diacetate (DCFH-DA) fluorescence dye, and 3,3’-diaminobenzidamine (DAB) were purchased from Sigma-Aldrich (St. Louis, MO, USA). All other reagents, including DA hydrochloride, DOPAC, and HVA were purchased from Sigma-Aldrich.

### 4.2. Preparation of the JSE and Standardization

JS was purchased from Jung Do Herbal Drug Company (Seoul, Korea) and a voucher specimen (KHUOPS-CMH001) was deposited in the herbarium at the College of Pharmacy, Kyung Hee University, Seoul, Korea. Extraction of JS was identical to that of reported before [25]. Briefly, JS (100 g) was soaked in 1 L of distilled water for 5 min at room temperature. Then, the extract was filtered, evaporated on a rotary vacuum evaporator, and lyophilized (yield; 0.05%). The powder was kept at 4 °C before use.

JSE was standardized according to our previously published method [25]. Briefly, JSE was standardized based on caffeic acid content determined using an Agilent 1100 high performance liquid chromatography (HPLC) system (Agilent Technologies Inc., Santa Clara, CA, USA) equipped with a quaternary solvent delivery system, an autosampler, and a DAD detector. J’sphere ODS-H80 columns (250 mm × 4.6 mm, 4 μm; YMC Co., Ltd., Kyoto, Japan) were used for analysis, and the mobile phases (A: CH_3_CN, B: 2% acetic acid in H_2_O) were 95%–60% B for 0–20 min, 60%–45% B for 5 min, and 45%–95% B for 5min. Chromatography was carried out in gradient mode using a flow rate of 1.0 mL/min at 30 °C and detection at 280 nm. The sample injection volume was 10 μL. Reference to the calibration curve was obtained with caffeic acid.

### 4.3. Primary Rat Mesencephalic Neuron/Glia Mixed Cultures and Treatment

Primary rat mesencephalic neuron/glia mixed culture system was generated as previously described [38,69]. The cell cultures were prepared from the ventral mesencephalons of 14-day embryos of timed pregnant Sprague-Dawley rats (Orient Bio., Osan, Korea). Briefly, after the mesencephalons were dissected, collected, and dissociated, the cells were seeded onto PLL pre-coated cover slips in 24-well plates at a density of 1.5 × 10^5^ cells/well in MEM supplemented with 6.0 g/L glucose, 2 mM glutamine and 10% FBS. Cultures were maintained in a water-saturated atmosphere of 5% CO_2_ at 37 °C. Seven-day-old cultures were used for treatment. The composition of the cells at the time of treatment was 45% astrocytes, 5% microglia and 50% neurons with 6% of the neurons being dopaminergic neurons in control conditions. The cells were treated with JSE (0.1 and 1 μg/mL) for 1 h and then with 15 μM MPP^+^ for an additional 23 h. After culture supernatants were collected separately, the treated cells were fixed with 4% paraformaldehyde (PFA; pH 7.3–7.4 values) or 30 min at room temperature for TH immunocytochemistry.

### 4.4. Assessment of MAO-B Activity and Expressions Levels in in Vitro System

In *in vitro* studies, MAO-B activity was measured using purified mitochondria from rat liver homogenates according to the previously described method [70]. Briefly, liver tissue (5 g) was homogenized 1:40 (*w*/*v*) in 0.3 M sucrose and centrifuged at 1000× *g* for 10 min. The supernatant was further centrifuged at 10,000× *g* for 30 min to obtain a crude mitochondrial pellet. The pellet was suspended in 4 mL of 0.3 M sucrose and was layered onto 40 mL of 1.2 M sucrose. A mitochondrial pellet was obtained by centrifugation at 53,000× *g* for 2 h. After a single wash in a potassium phosphate buffer, the mitochondria were suspended in 40 mL. To test for specific MAO-B activity, the rat liver homogenate was pre-incubated with 50 μM clorgyline, a selective MAO-A inhibitor, at 37 °C for 30 min. The mitochondria sample was divided into aliquots and stored at −70 °C until use. Tyramine (120 μL, 2.5 mM), an amine substrate, 40 μL of 1 mM vanillic acid with 0.5 mM 4-aminoantipyrine and 4 U/mL peroxidase, 40 μL of enzyme (rat liver homogenate), and 40 μL of JSE (0–1000 μg/mL) or ginkgo leaf (0–1000 μg/mL) or selegiline (0–100,000 μg/mL) were mixed. The mixture was measured using spectrophotometer at an absorbance of 490 nm at 37 °C. Absorbance values of the negative control curve were subtracted from the MAO-B positive control curve absorbance values. Three independent experiments were performed in triplicate.

### 4.5. Assessment of Intracellular ROS and NO Generation

ROS and NO generation were measured as previously described [38,69]. Intracellular ROS generation was measured using DCFH-DA fluorescence dye. DCFH-DA enters cells passively and is converted into non-fluorescent DCF-H, which reacts with intracellular ROS to form the fluorescent product DCF [49]. The formation of 2′,7′-dichlorofluorescein as the oxidized fluorescent derivative was monitored at an excitation wavelength of 488 nm and an emission wavelength of 525 nm. Also, the accumulated level of extracellular NO in culture supernatants was measured using the colorimetric reaction with the Griess reagent.

### 4.6. Animals

Animal maintenance and treatment were carried out in accordance with the Principles of Laboratory Animal Care (National Institutes of Health (NIH) publication No. 85-23, revised 1985), the Association for Assessment and Accreditation of Laboratory Animal Care system and the Animal Care and Use Guidelines of Kyung Hee University. Male C57BL/6 mice (7 weeks, 20–22 g) were purchased from Daehan Biolink Co., Ltd. (Eumseong, Korea). Animals were housed at an ambient temperature of 23 ± 1 °C, relative humidity of 60% ± 10% under a 12 h light/dark cycle, and were allowed free access to water and food. All experiments with mice were performed according to the protocols approved by the Institutional Animal Care and Use Committee of Kyung Hee University (KHP-2009-12-11; 29 December 2009).

### 4.7. Drug Administration

Mice were assigned to five groups: (1) control group (*n* = 15; intraperitoneally (*i.p.*) vehicle injected plus orally vehicle treated group); (2) MPTP group (*n* = 15; *i.p.* MPTP injected plus orally vehicle treated group); (3) MPTP + JSE 100 mg/kg/day group (*n* = 15; *i.p.* MPTP plus orally JSE treated group); (4) MPTP + selegiline 4 mg/kg/day group (*n* = 10; *i.p.* MPTP plus orally selegiline treated group); and (5) JSE 100 mg/kg/day group (*n* = 8; *i.p.* vehicle injected plus orally JSE treated group). JSE dissolved in normal saline was administered for 6 days consecutively. The control group received an equal volume of normal saline for the same duration. Acute-MPTP administration was generated as previously described [38]. On the third day of JSE treatments, MPTP at 20 mg/kg dissolved in saline was intraperitoneally injected four times at 2 h intervals. Vehicles of equal volume (0.25 mL) were given to the control group.

### 4.8. Behavioral Evaluation

#### 4.8.1. Locomotor Activity

The locomotor activity test is a useful method to measure poor gait and ambulation in mice PD model. A locomotor activity test carried out as reported before [71]. We performed the locomotor activity test on the 7th day after the last MPTP injection. All measurements were taken between 9 p.m. and 2 a.m. to avoid diurnal variation. The mice were placed in the testing chamber (40 cm × 25 cm × 18 cm) with black floors for 15 min adaptation, followed by a 60-min recording period using a computerized automatic analysis system (Biobserve GmbH, Bonn, Germany). The chamber was placed into adjustable frames equipped with seven infrared photocell beams. Ambulation was measured as the number of sequential breaks in two adjacent beams.

#### 4.8.2. Pole Test and Rotarod Test

The pole and rotarod test are models to measure bradykinesia and hypokinesia in mice PD model. The pole test and rotarod test were performed as the previously described [38,69]. We performed the pole test on the 7th day after the last MPTP injection. The mice were held on the top of the pole (diameter 8 mm, height 55 cm, with a rough surface). The time needed for the mice to climb down and place four feet on the floor was recorded as the time for locomotion activity (T-LA). Each trial had a cutoff limit of 30 s.

Additionally, we performed the rotarod test on the 7th day after the last MPTP injection. To assess sensorimotor coordination, the mice were evaluated with the rotarod test. The rotarod unit consists of a rotating spindle (diameter 7.3 cm) and five individual compartments to test five mice at a time. After twice daily training for two successive days (rotation speed 4 rpm on the first day and 20 rpm on the second day), the rotation speed of the test was increased to 25 rpm on the third day in a test session. The time each mouse remained on the rotating bar was recorded for three trials for each mouse, at a 5 min interval and a maximum trial length of 300 s per trial. Data were presented as mean time on the rotating bar over the three test trials.

#### 4.8.3. Brain Tissue Preparation

Brain tissue preparation was performed as previously described [38,69]. Briefly, on the 7 days after MPTP treatment, mice were perfused (outflow: 2.5 mL/min during 15 min) transcardially with 0.05 M PBS, and then fixed with cold 4% PFA (pH 7.3–7.4 values, outflow: 2.5 mL/min during 10 min) in 0.1 M phosphate buffer. Serial 30 µm-thick coronal sections were cut on a freezing microtome (Leica instruments GmbH, Nussloch, Germany) and stored in cryoprotectant at 4 °C until use for an immunohistochemistry (IHC) experiment. For MAO activity assay and HPLC analysis, the mice were decapitated and the striatum (ST) tissues were isolated and stored at −80 °C until use.

### 4.9. Measurement of Anti-Tyrosine Hydroxylase (TH)-Positive Dopaminergic Neurons and GFAP/MAO-B Co-Localizaiton

Fixed mesencephalic cells on coverslips or free-floating brain sections were rinsed with PBS before immunostaining, and then pre-treated with 1% H_2_O_2_ for 15 min to remove endogenous peroxidase activity. To determine TH immunoreactivity, every three sections (four per 12 sections) between Bregma −3.28 to −3.64 mm of each mouse were stained as the previously described method [38,69]. Cells or sections were incubated overnight at 4 °C with a rabbit anti-TH (1:2000 dilution). On the next day, they were incubated with a biotinylated anti-rabbit IgG (1:200 dilution) for 1 h, followed by incubation in ABC solution for 1 h. The peroxidase activity was visualized with DAB in 0.05 M tris-buffered saline (pH 7.6). Quantification of the effects on primary mesencephalic neurons or brain tissue sections was performed by counting TH-immunoreactive (TH-IR) cells in the SNpc and by measuring the optical density of TH-IR fibers in the ST using Image J software (Bethesda, MD, USA).

To examine GFAP/MAO-B co-localization, brain sections were washed with PBS and incubated with a rabbit anti-GFAP antibody (1:500 dilution). The sections were incubated with a goat anti-rabbit IgG Cy3 conjugate Alexa 594 (1:500 dilution) and then incubated with a goat anti-MAO-B antibody (1:500 dilution). After rinsing in PBS, sections were incubated with biotinylated anti-goat IgG (1:200 dilution) and then incubated with streptavidin-Alexa 488 (1:500 dilution) for 1 h, respectively. The images were photographed at 100× and 400× magnification using an optical light microscope (TH-positive photographs) or a fluorescence microscope (GFAP/MAO-B co-localized photographs) (Olympus Microscope System BX51; Olympus, Tokyo, Japan) equipped with a 20× objective lens. Data are presented as percentages of the control group values.

### 4.10. Assessment of Activity of MAO Isoforms in the in Vivo System

MAO-A and B activity were purified from ST of mouse brain homogenates using an MAO activity Assay Kit (Abnova, Taiwan), according to the previously described method [72]. To determine MAO-A activity, 1 mM *p*-tyramine substrate and a control with 0.5 μM MAO-A inhibitor clorgyline were used. The mixture was incubated for 10 min at room temperature for the inhibitor to block MAO-A activity. Five μL H_2_O_2_ 20 mM inhibitor with 10 mL H_2_O, working reagent, 50 μL assay buffer, 1 μL *p*-tyramine, 1 μL dye reagent and 1 μL HRP enzyme were mixed. Finally, they were incubated for 20 min in the dark and measured using the fluorescence intensity at 530 nm (excitation) and 585 nm (emission). To measure MAO-B activity, 1 mM *p*-tyramine and a control with 0.5 μM pargyline (MAO-B inhibitor) were used. The following procedure is the same as for MAO-A determination.

### 4.11. Measurement of Neurotransmitter Levels by HPLC

Measurement of neurotransmitter levels using HPLC was performed as the previously described method [71]. Briefly, the tissue contents of the neurotransmitter were measured using HPLC (Dionex Corp., Synnyvale, CA, USA) in combination with an electrochemical detecting system (ESA Coulochem III detection system, range 100 nA, potential −50 mV to +300 mV; ESA Inc., Chelmsford, MA, USA). The ST tissues were homogenized in 0.2 M perchloric acid. Homogenates were centrifuged for 20 min at 0 °C and 14,000× *g*. The supernatant was filtered through a 0.22 μm membrane, and an aliquot (10 μL in volume) of the resulting solution was injected into the HPLC pump. Chromatographic separation was performed using a C18 reverse-phase column (150 mm × 3 mm, 3 μm; Thermo Scientific, San Jose, CA, USA) at 25 °C, and data analyses were performed using Chromeleon^TM^ software (Version 6.40; Thermo Scientific, San Jose, CA, USA). The mobile phase, which had a pH of 4.0, consisted of 150 mM ammonium acetate, 140 μM ethylenediaminetetraacetic acid, 15% methanol, and 5% acetonitrile. The flow rate was maintained at 0.2 mL/min and the temperature of the column was 25 °C. DA, DOPAC, and HVA standards were prepared in 0.2 M perchloric acid and each sample concentration was adjusted with respect to the standard and quantified from a standard curve. The levels of DA, DOPAC, and HVA were calculated as nanograms per microgram of total protein which was determined with the Bradford protein concentration assay.

### 4.12. Statistical Analysis

All statistical parameters were calculated using GraphPad Prism 5.0 software (GraphPad Software Inc., San Diego, CA, USA). Values were expressed as the mean ± standard error of the mean (SEM). The results were analyzed by one-way analysis of variance (ANOVA) and post hoc multiple mean comparisons (Tukey’s HSD test).

## 5. Conclusions

In the present study, the polyphenol-rich aqueous walnut extract (JSE), protected dopaminergic neurons against MPP^+^ or MPTP-induced neurotoxicity as an MAO inhibitor with antioxidant activity. JSE also prevented depletion of striatal DA and its metabolites. Owing to these effects, JSE also ameliorated motor symptoms in a mouse model of PD. Taken together, these results indicate that JSE may be a potential candidate for the prevention or treatment of Parkinson’s disease.

## Supplementary Materials

Supplementary materials can be found at http://www.mdpi.com/1422-0067/17/1/108/s1.
