# Supplementary material for: In Vitro and in Vivo Neuroprotective Effects of Walnut (Juglandis Semen) in Models of Parkinson’s Disease
Source: Int J Mol Sci. 2016 Jan 15;17(1):108. doi: 10.3390/ijms17010108 (PMC4730349; doi:10.3390/ijms17010108)
Supplement: Supplementary file 1 [file ijms-17-00108-s001.pdf]

Supplementary Materials: *In Vitro* and *in Vivo* Neuroprotective Effects of Walnut (Juglandis Semen) in Models of Parkinson's Disease

Jin Gyu Choi, Gunhyuk Park, Hyo Geun Kim, Dal-Seok Oh, Hocheol Kim and Myung Sook Oh

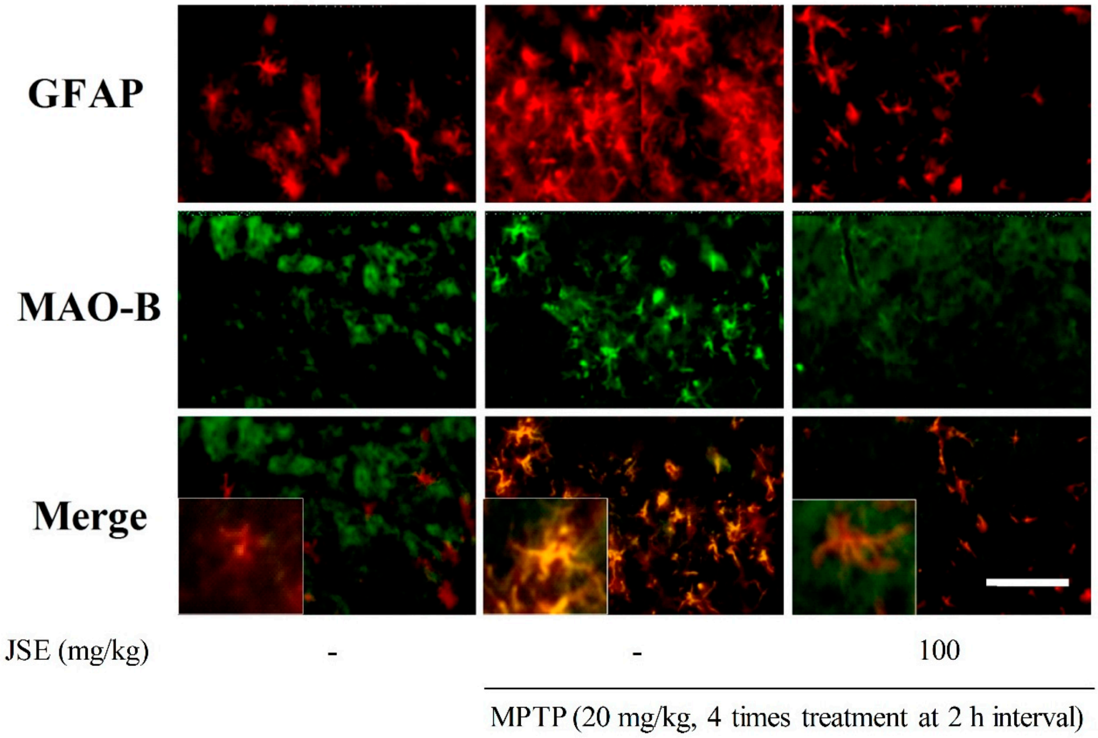

Figure S1. Effect of JSE on MPTP-induced glial fibrillary acid protein (GFAP) and monoamine oxidase B (MAO-B) inhibition in the substantia nigra pars compacta of mouse brain using GFAP and MAO-B immunohistochemical co-localization. Scale bar = 150 μm.
